# RETRACTION: Evaluation of Wound‐Healing Activity of Hydrogel Extract of *Sansevieria trifasciata* Leaves (Asparagaceae)

**DOI:** 10.1155/adpp/9783217

**Published:** 2025-11-21

**Authors:** Advances in Pharmacological and Pharmaceutical Sciences

RETRACTION: N. Yuniarsih, H. Hidayah, N. S. Gunarti, et al., “Evaluation of Wound‐Healing Activity of Hydrogel Extract of *Sansevieria trifasciata* Leaves (Asparagaceae),” *Advances in Pharmacological and Pharmaceutical Sciences*, 2023, https://doi.org/10.1155/2023/7680518.

The above article, published online on 29 August 2023 in Wiley Online Library (https://wileyonlinelibrary.com), has been retracted by agreement between the journal Editor‐in‐Chief, Kim Wei Chan, and John Wiley & Sons Ltd. The retraction has been agreed due to multiple concerns with Figure 5, which were initially raised on PubPeer [1]. Specifically,

• The 4 days/NC panel and the 2 days/15% HESt panel appear similar.
• The following panels appear similar:
  ◦ 8 days 15% HESt panel
  ◦ 8 days 25% HESt panel
  ◦ 16 days NC panel
• At 16 days, the 20% HESt and 25% HESt panels appear similar.
• At 0 days, the PC and 20% HESt appear similar if rotated 90°.

Additionally, on further assessment by the Editorial Board, inconsistencies with the methodology were identified. Specifically,

• The extract was initially prepared using 96% ethanol, but the extract used for HPLC quantification was obtained through a completely different solvent system (70% methanol) and extraction procedure.
• The HPLC chromatogram provided appears to lack proper methodological optimisation prior to quantification.

The authors were asked to respond to the concerns with the figures, however could not provide the raw data and original images.

The authors disagree with the retraction.
